# Monthly Summary

**Published:** 1898-11

**Authors:** 


### Monthly Summary.

---

TAKING IMPRESSIONS.—With reference to taking an impression in plaster for a partial plate, or in taking a full impression of the teeth, I scarcely know who is the author of that method. But I have learned it was Dr. Angle's method of taking an impression of the teeth, which consists of using a clean impression cup evenly filled with plaster, leaving but little surplus. Wait till your plaster has set hard; then slip the cup off, it having been oiled slightly, leaving the plaster all in the mouth. Two grooves are then cut in the hardened plaster on a line parallel with the cuspid teeth, not cutting quite through. Then, with a quick pry with a pointed spatula or knife, the anterior piece is wrenched loose. The lateral pieces are broken off with the thumb and finger. The large piece covering the roof of the mouth may readily be worked loose. Then you have four pieces which are easily united.—G. D. Sitherwood, in Illinois State Society.

---

CARIOUS DENTIN.—I remove everything except sound dentin, regardless of pulp exposure. If the pulp is that near exposure I destroy it, remove it, and fill the canals. If I left decayed dentin in proximity to the pulp I should expect trouble to follow.—Dr. L. M. Corwardin, in *Dental Brief.*

---

GOLD-SHELL CROWN—I would not discard the gold-shell crown for molars and bicuspids, so there is no other kind of crown which is so easily made, and, all things considered, gives such satisfaction.—W. H. Bailey, in *Dental Weekly.*

ICHTHYOL.—Some time since a case presented with an abscess, which seemed to be coming to a point externally. The abscess had started from the root of a first molar on the lower jaw, and had burrowed down through the swollen tissue until only a thin layer of solid tissue existed between the forming pus and the outer surface, the thinnest point being just under the submaxillary gland. The abscess was thoroughly lanced, and the cavity freed of pus by injections of three per cent pyrozone, but at each subsequent sitting the pus had burrowed further down and the layer of solid tissue was perceptibly thinner.

It was now decided to use a bandage in conjunction with an ichthyol poultice, to support the parts and try to prevent an external opening. Accordingly a small piece of linen was cut to cover the affected region and smaared thickly with ichthyol.

This being stuck on, the parts were tightly bandaged by passing a bandage over the head and under the chin, supporting the parts and holding the poultice in place. The results were most gratifying, besides the abortive effect of the ichthyol on the abscess; after a few hours it had become dried out, lerving stiff, hard support, which had been exactly moulded to the contour of the parts. It seemed the only way by which the external breaking of the abscess could be avoided.

Later we notice that ichthyol has been employed by Dr. H. Foria in treating pyorrhea and painful receded gums. In the former case he uses it full strength in the pockets, and afterwards syringes with a fifty per cent. solution. He has also used it as a tampoon in the sockets of extracted teeth to prevent nemorrhage.—H. H. Johnson, in Dental Weekly.

---

THE BEST STYPTIC.—What is the best styptic? Hot water. Keep it up and you will surely conquer. Loot at the washerwoman's hands after a day's work in the tub of hot water; note how pale and bloodless, shriveled and shrunken they are and learn a lesson.—J. Y. Crawford, in *Dental Brief.*

USING CLAMPS.—At a recent meeting of the Chicago Dental Society Dr. Fernandez described a method of using clamps on teeth which, I think, if universally used, would get rid of the uncomfortable sensation around the tooth, and the tipping forward, pressing into the gum at anterior portion. He suggested that the points or sharp edges which ordinarily project and cut into the tooth structure be filed away and a small rubber tube be placed over the clamp, leaving the ends of the rubber tubing a little longer than the clamp, to prevent it from tipping, which it so often does, and is very inconvenient and causing great pain. I think the suggestion is a good one, and if used would be beneficial in every-day practice. Many dentists do not use the clamp on account of the saliva clogging the mouth. This trouble can be obviated by the saliva pump attached to the fountain cuspidor. The clamp should not be used unless where it is difficult to keep the dam away from the tooth, and where the cavity is situated on the buccal surface, answers a good purpose to keep gum and rubber away from tooth.—I. B. Crissman, in Illinois State Society.

---

SOLDERING TEETH.—Great care ought to be taken to prevent the borax from getting between the tooth and the backing, or on the cutting edge of the tooth. The borax sticks to the tooth, and if one grinds it off, it generally chips away and brings a piece of the tooth with it. If the borax is allowed to get on to the tooth, it very often results in small cracks running down the tooth. See that the teeth are quite clean before investing; if hard wax is allowed to remain on, the tooth will discolor when soldering. It is better to scald the plate with hot water, when invested, to remove the wax and make everything quite clean. Before investing, a slight space must be made between the teeth to allow for the contraction of the solder, which is inclined to bring the teeth inwards, and if the teeth are touching before investing, the conraction often fractures them.—F. Mackenzie, in "British Journal Dental Science."

—DESTROYING PULPS WITHOUT ARSENIC.—The method is exceedingly simple and very effective. It is not orfginal at least as I now practice it. Formerly I used the method to do away with the pain which still remained in the small particles of the pulp which were left in the roots, the greater part of the pulp having been devitalized by arsenic. However, as a method has been lately discovered to devitalize the pulp as easily as I did the remaining particles, I resolved to try my method on the pulp, and it worked like a charm. Here it is: Dry the cavity out after having removed as much of the debris as practical without giving a great deal of pain; then take a piece of soft spunk, dip it in alcohol (absolute alcohol is the best) and then dip the alcohol-laden spunk in crystals of muriate of cocaine, place X in the bottom of the cavity and press a piece of unvulcanized rubber against it quite hard for from one to three minutes; then take out and remove the remaining layers of decay till you thoroughly expose the pulp, and repeat the operations, when you will find the pulp has lost all sense of feeling and you can remove it without the slightest pain.

Be careful to remove all the pulp before filling; as sensitiveness does not return for from ten to fifteen minutes. A. J. McDonagh, in *Dominion Dental Journal.*

---

COUNTING BLOOD CORPUSCLES.—An instrument for counting blood corpuscles has been invented by Dr. Judson Deland, of Philadelphia. A finely graduated tube containing a quantity of blood is revolved at a speed of one thousand revolutions a minute. By force of gravity the corpuscles divide and form on the side of the tube in easily traceable divisions of red corpuscles, white corpuscles and serum.—"Dental Digest."

---

TRIMMING VULCANITE.—Sharpened chisels dipped in oil trim vulcanite as easy as cutting soft chocolate.—Dominion Dental Journal.

OPERATIVE AND PROSTHETIC POINTERS.—It not infrequently happens that the operator accidentally applies carbolic acid to the lips of his patient, and unless he is able to relieve the immediate suffering and the eschar which invariably results, he is likely to bring upon himself the unpleasant phrase of, "He is a careless, hustling dentist." Now if you have had the misfortune of getting the carbolic acid in touch with the lips, you can avoid all difficulties by bathing the burned surface with vinegar and all unpleasant after-effects will be avoided.

Resorcin may be employed as a local anesthetic. Resorcin, as we all know, has the properties of an antiseptic, since it is a derivative of carbolic acid and possesses many of the powers of the latter. Its qualities as a local anesthetic, however, are of considerable interest. In cases of stomatitis there is great need for such a remedy, and the mucous membrane can be painted with a mixture of resorcin 5 parts, water 30 parts. On account of its less irritating properties, it is considered one of the best antiseptic agents to be employed in the oral cavity. In diphtheria or other diseases causing a sloughing sore, no better results can be had than applying a mixture of resorcin to the affected portion. It accomplishes all that can be expected of carbolic acid and does it in a painless manner. For dental ulcerations it can be used in the form of a liquid; in case it is to be used as a dressing for wounds it should be used in crystal form,—"Digest."

---

TO GIVE FINE FINISH TO GOLD.—After the scratches have been removed with pumice stone, nothing is so effectual as oxide of zinc on a brush wheel. It leaves a beautiful, lustrous polish, and does not soil the hands.—H. H. Johnson, in *Dental Weekly.*

---

SOAP ON DAM AND DISK.—A little soap on the dam about the holes will make it pass between crowded teeth with ease. Soap on the edge of a sandpaper or cuttlefish disk will prevent it from catching to the palm.—T. P. Hinman, in *Dental Weekly.*

GUTTA-PERCHA AS A RETAINER OF CROWNS AND BRIDGES.—Every practitioner should be prepared to attain definite and satisfactory results in the setting of post crowns and bridges. It not infrequently occurs that the operator is obliged to remove a recently positioned crown or bridge; this undesirable operation is usually caused by pericemental troubles or possibly apical disturbances, and all practitioners are familiar with the hard task of eliminating the cement which is generally employed as a retainer. In repairing bridges we are also obliged to remove the entire bridge, and the displeasure to both patient and operator is well known. But if the crown or bridge be retained by gutta-percha the removal need not give us trouble since, if we grasp the crown or pillar of the bridge with a hot forcep, the heat of the latter is readily conveyed to the gutta-percha, which, yielding to the warmth, admits of an easy detachment of the denture. In positioning a crown having gutta-percha as a retainer, it is wise to inquire of the patient whether any pain was felt; if so, the gutta-percha closely filling the root-canal has forced air through the apical foramen and this has induced the pain. Be certain to have but a sparing amount of gutta-percha on the post, and ascertain the exact amount by previous trial and trimming. Not only have we by this method accomplished retention of the bridge, but we have anchored it with a substance which is not acted upon by the dirt or fluids of the mouth.—Translated by Dr. B. J. Cigrand from "Zahnartzliches Wachenblatt," Oct., 1897.

---

CELLULOID MATRICES.—Dr. Schultz, of Kansas, says celluloid makes the very best matrix for cement filling; that the surface in contact with the matrix will be perfectly polished and finished when the matrix is removed. Thin sheets of celluloid can be had of photographers.—"Dental Weekly."

---

NOT HARD.—It is not hard to distinguish the reading dentist from the non-reading dentist, when they are heard to speak at a meeting.—"Dental Weekly."

TO DEPOSIT PASTE IN CAVITY.—In capping small exposures, or when putting cement into an excessively sensitive cavity, I am accustomed to use a stiff paste of white oxid of zinc and oil of cloves next the pulp or dentin. I found it difficult to carry this paste to the exact spot and leave it there, owing to its strong inclination to stick to the instrument used in depositing it. Finally I discovered almost by accident that if the paste was made into a little ball it could be carried to the cavity and placed exactly where needed, or spread over the exact amount of surface required, by using a bit of spunk in the pliers, as carrier and spreader, the spunk coming away almost clean, or at most with only a little oil upon it. This is a little point, but it has given me a great amount of satisfaction.—J. H. Hughes, D. D. S., Goshen, Ind., in "Dental Review,"

---

FOR POLISHING PLATE WORK.—For quickly cutting down or rough polishing rubber or gold plate work, I find no better method than with the porte polisher, run with the dental engine.

By removing the nut, a strip of three-fourths of an inch wide sandpaper or emery cloth may be slipped in the slot, and run as the same would be in a regular polishing lathe, only, this device run by the engine offers a more delicate result, as the instrument and handpiece are manipulated on the plate instead of the plate being manipulated against the polisher, as in the case with the lathe. The difference makes the result very different, and the operation almost a pleasure.—Dr. T. L, Smith, in *Items*.

---

PYORRHEA.—I have been very successful in the treatment of pyorrhea alveolaris. In cases where other gentlemen have failed, I extract.—Dr. Wm. Carr, in "Cosmos."

